# Analysis of preoperative and postoperative depression and anxiety in patients with lumbar disc herniation with radiculopathy treated with percutaneous transforaminal endoscopic discectomy

**DOI:** 10.3389/fpsyt.2024.1460274

**Published:** 2024-10-25

**Authors:** Yatao Wei, Hailun Huang, Kui Sun, Heng Gao, Zhenwen Cao, Bin Zhang, Junzhe Wu, Yongai Liu

**Affiliations:** Zhongshan Hospital of Traditional Chinese Medicine Affiliated to Guangzhou University of Traditional Chinese Medicine, Zhongshan, Guangdong, China

**Keywords:** lumbar disc herniation with radiculopathy, percutaneous transforaminal endoscopic discectomy, depression, anxiety, prognosis

## Abstract

**Objective:**

This study aims to examine the mental health of patients with lumbar disc herniation with radiculopathy (LDHR) and to evaluate the effects of percutaneous transforaminal endoscopic discectomy (PTED) on their mental well-being.

**Method:**

This study included 114 LDHR patients who received PTED in the Spinal Orthopedics Department of Zhongshan Hospital of Traditional Chinese Medicine between May 2022 and May 2023. The study population was stratified into two groups according to the presence of preoperative anxiety and depression: Group A comprised patients with such symptoms, and Group B included the remainder. Patients were assessed using the Hospital Anxiety and Depression Scale (HADS), the Visual Analog Scale for Pain (VAS), and the Oswestry Disability Index (ODI) at preoperative and final follow-up visits. Evaluations were performed using the Hospital Anxiety and Depression Scale (HADS), the Visual Analog Scale, and the Oswestry Disability Index (ODI) at the time of preoperation and during the last follow-up.

**Results:**

Among the 114 LDHR patients who obtained complete follow-up, fifty-four had anxiety/depression symptoms before surgery, which accounted for a incidence of 47%. Both Group A and Group B patients showed significant improvement in all evaluation indicators after PTED, but the overall prognosis of Group A was worse than that of Group B.

**Conclusion:**

PTED can effectively improve patients’ pain, functional activities and mental health, which has a positive impact on patients’ quality of life, and there is a significant correlation between patients’ preoperative mental health and prognosis.

## Introduction

Lumbar disc herniation with radiculopathy (LDHR) is among the common causes of substantial quality of life decline, primarily due to the displacement of disc material beyond its normal limits, which compresses nerve roots and results in lower limb pain, weakness, and sensory disturbances (1, 2). Initial management of LDHR typically emphasizes conservative methods; however, surgical intervention is necessary when conservative measures prove ineffective. Studies have shown that surgical treatment can notably alleviate the symptoms and enhance the quality of life for patients with LDHR (3). In the past, open surgery was the standard treatment for LDHR. However, with the advancement of minimally invasive spinal surgery, percutaneous transforaminal endoscopic discectomy (PTED), which is noted for its minimal invasiveness, rapid recovery, and proven efficacy, has increasingly emerged as one of the commonly surgical approaches for managing LDHR patients worldwide (4).

LDHR typically adversely affects patients’ daily and recreational activities, which can lead to a substantial psychological strain on the individuals affected. Research indicates that individuals with preoperative anxiety and depression symptoms experience suboptimal clinical outcomes and satisfaction levels following lumbar surgery (5–8). Moreover, there is a noted correlation between mental health status and the severity of symptoms in patients (9), with those experiencing poorer mental health being more sensitive to their symptoms. Therefore, the improvement of patients’ preoperative mental health is a crucial aspect of developing a surgical strategy. Nevertheless, there is a limited body of research examining the preoperative and postoperative mental health status of patients who undergo PTED for LDHR. The aim of this study is not only to investigate the incidence of preoperative depression or anxiety symptoms in LDHR patients, to facilitate the assessment of the impact of PTED on patients’ mental health. Moreover, we have also studied the influence of preoperative mental health status on prognosis, to provide some reference for clinical treatment planning.

## Patients and methods

### Patients

**Figure 1:**
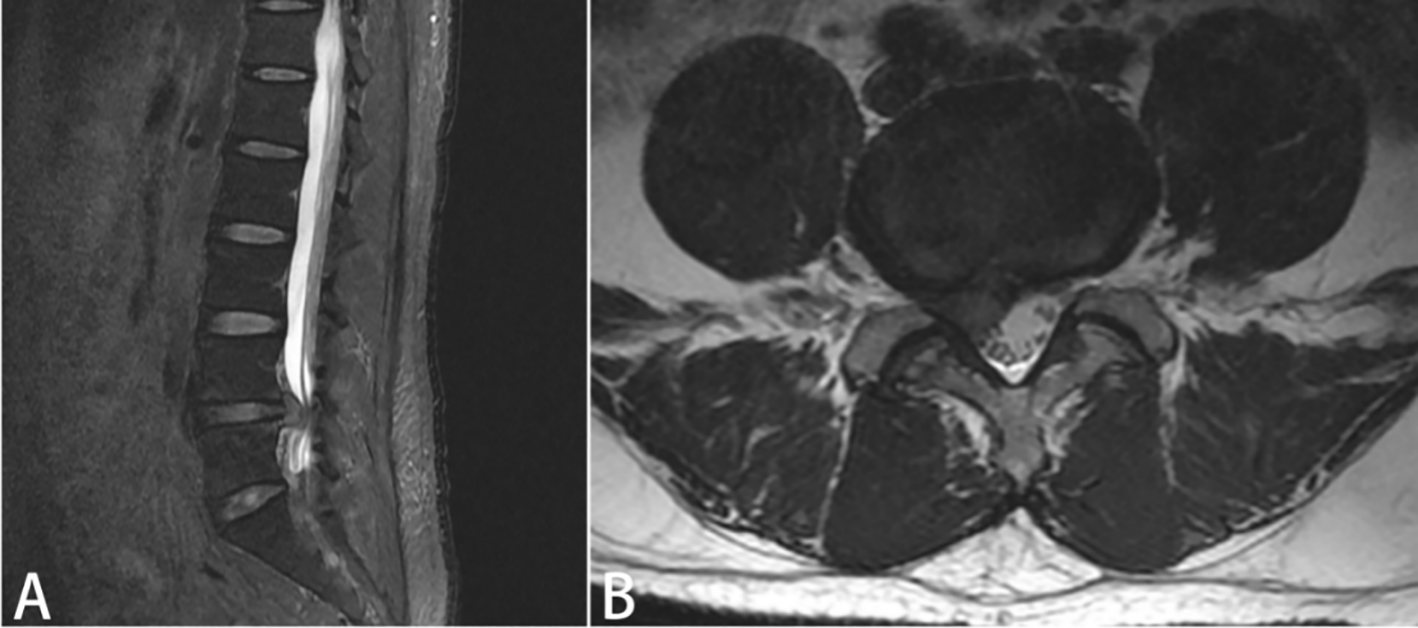
The patient’s preoperative lumbar MRI **(A)**: Sagittal view, **(B)**: Coronal view) reveals a right paracentral herniation at the L4/5 intervertebral disc, with compression of the right nerve root.

**Figure 2:**
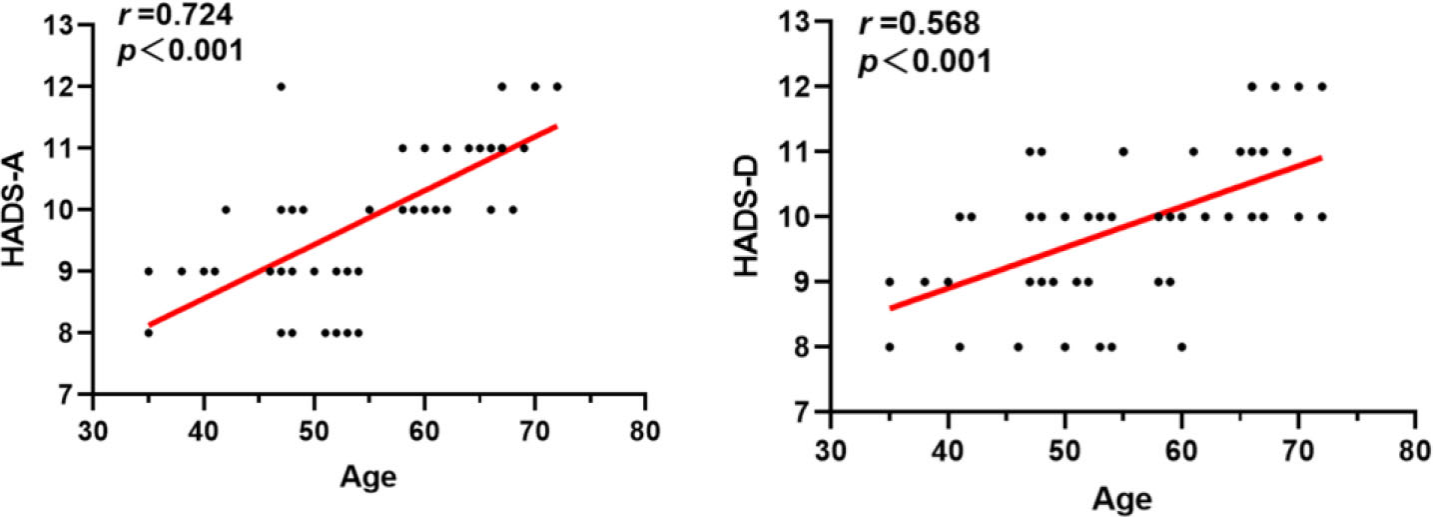
Analysis of the correlation between age and preoperative anxiety/depression symptoms.

This study has been approved by the Ethics Review Committee of Zhongshan Hospital of Traditional Chinese Medicine(No:2024ZSZY-LL-KY-019). We retrospectively reviewed patients treated with percutaneous transforaminal endoscopic discectomy in the Spinal Orthopedics Ward of Zhongshan Hospital of Traditional Chinese Medicine between May 2022 and May 2023. Informed consent was obtained from all patients. The inclusion criteria were as follows (1): Spinal cord compression verified by MRI or CT, accompanied by distinct lower limb radicular pain symptoms (**Figure 1**); (2) Individuals who have failed to respond to at least 6 weeks of systematic conservative treatment (3); (3) single-segment disc herniation with radicular symptoms aligned with the imaging-confirmed segment. (4) Has the ability to independently complete questionnaires with language and thinking skills. The exclusion criteria were as follows: (1) A history of previous back surgery; (2) Multisegmental lumbar disc herniation; (3) Patients with severe osteoporosis, arthritis, spinal infections, spondylolysis, and other diseases; (4) Patients who have experienced traumatic events affecting their mental and psychological well-being.

### Methods

We contact patients through WeChat (Tencent, Shenzhen, China) or by phone, engage in face-to-face communication with them, and complete the questionnaire. During preoperative evaluations and at the final follow-up, the Oswestry Disability Index (ODI) and the Visual Analog Scale (VAS) for pain assessment are utilized to evaluate the patient’s pain levels and overall functional status. The Oswestry Disability Index (ODI) is a widely recognized and validated pain and functional assessment scale, with a score of 0 indicating no symptoms and a score of 100 indicating complete disability (10). Visual Analog Scale (VAS) is composed of a 100-mm line and is capable of accurately evaluating the patient’s pain intensity (11). We employ the Hospital Anxiety and Depression Scale (HADS) to evaluate patients for anxiety and depression at both preoperative stages and during the final follow-up assessment (12, 13). The HADS is composed of two subscales, the Anxiety subscale (HADS-A) and the Depression subscale (HADS-D), each containing 7 items. Items are rated on a 4-point scale from 0 to 3, with each scale having a score range of 0 to 21. A score of 8 or above suggests the presence of some level of anxiety or depression symptoms.

We divided patients with complete follow-up data into two groups: Group A (with preoperative anxiety/depression symptoms) and Group B (without preoperative anxiety/depression symptoms), to better understand the impact of mental health status on the prognosis of patients with PTED. All patients were anesthetized and operated on by the same group of doctors. The surgical procedure involved: After epidural anesthesia, a working sheath was inserted under fluoroscopic guidance using the C-arm, followed by the insertion of the endoscope to remove the herniated nucleus pulposus tissue. After the nerve was fully decompressed, the surgery was completed. All patients were then instructed on a specific rehabilitation exercise regimen following the operation.

### Statistical analyses

The data were analyzed with SPSS 27.0 software (IBM, New York, USA).The Kolmogorov-Smirnov test was employed to evaluate the normality of the data distribution. Data that conform to a normal distribution or are approximately normally distributed should be analyzed using t-tests, whereas non-parametric tests should be employed for data that do not meet these criteria. An independent samples t-test was employed to assess the differences in age, disease duration, and follow-up time across the two patient groups. A paired samples t-test was used to evaluate the differences between preoperative and final follow-up results within the group, with a significance level of P<0.05 indicating a statistically significant difference. The independent samples t-test was applied to assess the disparities in evaluation criteria between the two groups of patients at preoperative and final follow-up stages. For patients with anxiety/depression, an independent samples t-test and Pearson correlation analysis were used to evaluate the correlation between gender, age, postoperative improvement, and psychological state.

## Results

### General condition of the patient

A total of 116 patients were included in the study, with 114 patients having complete follow-up. The general information of the patients is presented in **Table 1**. Among them, 54 (47%) patients had symptoms of anxiety or depression before surgery, including 23 males and 31 females. Sixty patients (53%) did not exhibit symptoms of anxiety or depression before surgery, including 35 males and 24 females. There were no statistically significant differences in age, gender, duration of illness, follow-up time, surgical segments, and operation duration between the two groups (**Table 1**). In the perioperative period, we routinely administer oral non-steroidal anti-inflammatory drugs to relieve pain and ensure patient comfort. Concerning postoperative complications, none of the patients experienced severe issues like vascular or nerve damage. In group A, there were 3 cases and in group B, 5 cases of intraoperative dural sac rupture; however, because the dural tear was small, direct closure of the skin incision was conducted, and no notable discomfort was reported postoperatively

**Table 1:** General characteristics of the patients.

	Group A(n=54)	Group B(n=60)	P value
Sex,n(%)			
Male	23(43%)	35(57%)	0.093
Female	31(57%)	25(43%)	
Age (years)	56.00 ± 10.15	53.17 ± 8.18	0.106
follow-up time (months)	14.41 ± 1.22	14.00 ± 1.24	0.080
Duration of Illness (months)	32.52 ± 9.74	29.18 ± 14.57	0.150
Surgical Segment			
L3/4	4	3	0.866
L4/5	27	31	
L5S1	23	26	
operation time (minutes)	110.39 ± 12.85	113.20 ± 13.63	0.636
HADS-A (preop)	9.96 ± 1.23	4.77+1.35	<0.001
HADS-D (preop)	9.91 ± 1.12	4.98 ± 1.20	<0.001

### Analysis of the differences between preoperative and final follow-up assessment indicators

The 114 patients were followed up for a period ranging from 12 to 16 months. Throughout the follow-up period, there were no complications such as wound infections or lumbar instability observed in either group. The evaluation criteria for patients in Group A showed marked improvement after surgery, compared to their preoperative status(P<0.05, **Table 2**). The HADS-A score decreased from 9.96 ± 1.23 before surgery to 4.98 ± 1.07 after surgery, and the HASD-D score dropped from 9.91 ± 1.12 before surgery to 5.02 ± 1.28 after surgery. The VAS score decreased from 53.33 ± 6.69 before surgery to 23.89 ± 2.92 after surgery. The ODI score decreased from 60.07 ± 4.61 before surgery to 25.93 ± 3.75 after surgery. All comparisons were statistically significant (P < 0.001).

**Table 2:** Changes in evaluation indicators for patients in Group A before surgery and at the final follow-up.

Evaluation indicators	VAS	ODI	HADS-A	HADS-D
preoperative	53.33 ± 6.69	60.07 ± 4.61	9.96 ± 1.23	9.91 ± 1.12
final follow-up	23.89 ± 2.92	25.93 ± 3.75	4.98 ± 1.07	5.02 ± 1.28
t value	31.91	54.42	27.94	24.35
P value	P<0.001	P<0.001	P<0.001	P<0.001

In Group B, the HADS-A score decreased from 4.77 ± 1.35 before surgery to 2.80 ± 1.36 after surgery, and the HASD-D score dropped from 4.98 ± 1.20 before surgery to 2.87 ± 1.65 after surgery. The VAS score decreased from 60.45 ± 3.86 before surgery to 16.53 ± 1.99 after surgery. The ODI score decreased from 58.37 ± 3.48 before surgery to 17.98 ± 1.94 after surgery (P < 0.001, **Table 3**).

**Table 3:** Changes in evaluation indicators for patients in Group B before surgery and at the final follow-up.

Evaluation indicators	VAS	ODI	HADS-A	HADS-D
preoperative	60.45 ± 3.86	58.37 ± 3.48	4.77 ± 1.35	4.98 ± 1.20
final follow-up	16.53 ± 1.99	17.98 ± 1.94	2.80 ± 1.36	2.87 ± 1.65
t value	77.71	84.83	18.52	8.25
P value	P<0.001	P<0.001	P<0.001	P<0.001

### Correlation analysis of psychological status

Regarding the relationship between mental health and outcomes, the study revealed a statistically significant difference (p < 0.05) in postoperative evaluation metrics between the two groups (see **Table 4**), and that the prognosis of patients in Group A was worse than that of patients in Group B. Furthermore, we observed that preoperative VAS scores in group A were lower than those in group B, while ODI scores in group A were higher than those in group B. (P < 0.05, **Table 5**).

**Table 4:** Correlation analysis between psychological state and prognosis.

Evaluation indicators	VAS	ODI	HADS-A	HADS-D
t value	15.56	13.98	9.42	109.68
P value	P<0.001	P<0.001	P<0.001	P<0.001

**Table 5:** Correlation analysis between psychological state and preoperative VAS, ODI scores.

Evaluation indicators	VAS	ODI
groups t value	-6.86	2.21
P value	P<0.001	P<0.05

For the patients in Group A, we conducted a study to explore the correlations between gender, age, the improvement of postoperative evaluation indicators, and mental health status. Our findings indicate that there was no significant correlation between gender and preoperative anxiety/depression levels in Group A patients (p > 0.05, **Table 6**). However, there was a significant correlation between age and preoperative anxiety/depression levels (P < 0.001, **Table 6**), and age was positively correlated with preoperative anxiety/depression levels (**Figure 2**). Additionally, we found that the levels of preoperative anxiety and depression did not affect the degree of improvement in postoperative VAS and ODI scores (All P > 0.05, **Table 7**).

**Table 6:** Correlation analysis between gender, age, and preoperative psychological state in group A patients.

Evaluation indicators	HADS-A	HADS-D
Sex t value	1.79	1.84
P value	P>0.05	P>0.05
Age r value	0.724	0.568
P value	P<0.001	P<0.001

**Table 7:** Correlation analysis between postoperative improvement and preoperative psychological state in group A patients.

Evaluation indicators	VAS	ODI
Anxiety r value	0.115	0.204
P value	P>0.05	P>0.05
Depression r value	-0.015	0.25
P value	P>0.05	P>0.05

## Discussion

Lumbar disc herniation often (LDH) leads to chronic back and lumbar pain, with severe cases potentially resulting in disability (14). Particularly in patients with lumbar disc herniation accompanied by radiculopathy, the severity of clinical symptoms and injury is more pronounced, including severe pain, limited mobility, and even abnormalities in bowel and bladder function (15–17). Previous studies have reported that patients with LDH (Lactate Dehydrogenase) who have sciatica are more likely to experience symptoms of anxiety and depression compared to those without sciatica (18). Furthermore, there is a certain correlation between psychological status and sciatica as well as functional activity (19). For patients with lumbar disc herniation accompanied by radiculopathy, PTED is a safe and effective method to improve the patient’s lower back and leg pain, as well as their daily functional activities (20). However, in current clinical practice, the influence of psychological factors is rarely considered when formulating surgical plans.

Gadjradj et al. treated lumbar disc herniation with a less invasive PTED method, and the study found that, at 12 months, patients in the PTED group had a greater reduction in leg pain compared to those undergoing open microscopic lumbar discectomy (20). Yusen Dai et al. reported that following PTED, the mean ODI score decreased from 41.41 ± 4.53 preoperatively to 26.21 ± 3.16 at the final follow-up visit (21). Our study found that both groups of patients showed significant improvement in VAS and ODI scores after PTED. Additionally, we assessed the psychological well-being of patients who underwent PTED and found that, when compared to their preoperative scores, both the HASD-A and HASD-D scores for both groups showed a significant improvement post-PTED. Moreover, during the follow-up period, no patients from either group exhibited complications related to wound infection, lumbar instability, or similar issues. Therefore, we believe that PTED can be a safe and effective treatment for patients with lumbar disc herniation with radiculopathy.

This study included a total of 114 patients, with 47% of the patients having poor mental health status prior to surgery. Compared with other chronic diseases (such as hip osteoarthritis, knee osteoarthritis, breast cancer, diabetes), patients with lumbar disc herniation with radiculopathy (LDHR) have a higher prevalence of preoperative depressive and anxiety symptoms (22–24). Lebow et al. have found a significant correlation between preoperative anxiety/depression and the prognosis of microdiscectomy (25). Theologis et al. report that preoperative depressive symptoms significantly impact the clinical outcomes of adult spinal deformity surgery two years postoperatively (26). Regardless of whether it is pain, functional performance, or mental health, patients who have anxiety or depression before surgery tend to have a worse recovery than those without these symptoms prior to their surgery. Previous research has shown that patients’ mental health status is associated with disability indices and quality of life (27, 28). This study shows that there is a correlation between patients’ mental health status and the level of preoperative pain as well as the preoperative disability index. Therefore, it is necessary to optimize the mental health of patients with poor preoperative mental health status to help them achieve the best clinical outcomes.

Our research on Group A patients with poor mental health has found that there is no significant correlation between gender and preoperative anxiety/depression. Studies report states that the risk of anxiety/depression in women is twice that of men, and female patients often experience more severe and longer-lasting symptoms than male patients (29–31). The discrepancies between these studies and the conclusions of this study may be due to differences in the diseases of the study participants and cultural-geographical variations. Different types of diseases may manifest differently in terms of mental health status. Moreover, the influence of cultural background on mental health should not be overlooked; in Eastern cultures, emotional expression is often more restrained, whereas Western cultures may be more inclined to openly express anxiety and depression. This may result in differences in emotional assessments among patients from different regions (32, 33). Secondly, some studies utilized different scales than those used in the present research. Commonly used scales for assessing anxiety and depression in clinical practice include the SAS, SDS, pain-anxiety simulation scale, and the Hamilton anxiety and depression scales. Each of these scales emphasizes different aspects of the patients’ psychological state, which may lead to variations in the survey results (34). This study also found that there is a significant correlation between the age of patients in Group A and their preoperative anxiety/depression levels. Research indicates that engaging in regular physical activity can significantly decrease the incidence of depression (35). Elderly individuals, who may engage less frequently in physical activities, might experience poorer mental health. Furthermore, studies suggest a positive correlation between the severity of anxiety and depression and age (36, 37). This study found that group A patients still experience high levels of anxiety and depression postoperatively, which we believe may be related to the following factors: Firstly, although PTED can alleviate radiculopathy symptoms, some patients may still experience chronic pain or functional impairments, which are closely related to mental health issues. Secondly, individual differences such as psychological resilience and social support may also affect postoperative emotions. Additionally, the postoperative recovery process may be accompanied by psychological stress, and the gap between patients’ expectations for recovery and actual outcomes may exacerbate feelings of anxiety and depression. In our future research, we will further analyze these factors to gain a more comprehensive understanding of the psychological state of patients with anxiety/depression postoperatively. Although there is a certain correlation between mental health status and prognosis, the levels of preoperative anxiety and depression do not affect the extent of improvement in various postoperative evaluation indicators. Therefore, in clinical practice, it is necessary to assess the mental health status of elderly LDHR patients in order to develop personalized treatment plans for these individuals.

This study not only investigated the clinical efficacy of PTED in treating LDHR but also explored the correlation between patients’ mental health status and prognosis, aiming to provide a more comprehensive diagnostic and treatment service for patients to help them achieve better clinical outcomes. However, this study has certain limitations, as it included only 114 LDHR patients, which is a relatively small sample size. In addition, this is a retrospective study conducted at a level III A comprehensive Chinese medicine hospital. The results may not be applicable to patients treated in other hospitals. To further understand the association between mental health status after PTED and clinical outcomes, we need a large-scale, multi-center, long-term follow-up randomized controlled trial. Secondly, this study only examined the correlation between anxiety/depression and prognosis, but it is possible that other mental health conditions may also influence the prognosis.

## Conclusion

PTED is a safe and effective treatment option for LDHR patients. LDHR tends to trigger anxiety and depression symptoms in patients, with older patients exhibiting higher levels of anxiety and depression compared to younger patients. These psychological factors can influence patient outcomes. The severity of preoperative anxiety and depression symptoms is not associated with the degree of postoperative pain relief and improvement in functional activities.

## Data Availability

Publicly available datasets were analyzed in this study. If needed, information can be obtained from the corresponding author.
